# TRIM45 aggravates microglia pyroptosis via Atg5/NLRP3 axis in septic encephalopathy

**DOI:** 10.1186/s12974-023-02959-8

**Published:** 2023-11-30

**Authors:** Xuliang Huang, Changzhou Ye, Xinyu Zhao, Yao Tong, Wen Lin, Qingqing Huang, Yuhao Zheng, Junlu Wang, Anqi Zhang, Yunchang Mo

**Affiliations:** 1 Department of Anaesthesia, The First Affiliated Hospital of Wenzhou Medical University, Wenzhou, Zhejiang China; 2 Provincial Key Laboratory of Immune Regulation and Immunotherapy, School of Laboratory Medicine and Biotechnology, Southern Medical University, Guangzhou, Guangdong China

**Keywords:** TRIM45, NLRP3, Pyroptosis, Sepsis-associated encephalopathy, Microglia

## Abstract

**Background:**

Neuroinflammation mediated by microglial pyroptosis is an important pathogenic mechanism of septic encephalopathy (SAE). It has been reported that TRIM45 is associated with tumours and inflammatory diseases. However, the role of TRIM45 in SAE and the relationship between TRIM45 and microglial pyroptosis are unknown. In this study, we found that TRIM45 played an important role in regulating microglial pyroptosis and the molecular mechanism.

**Methods:**

SAE was induced by intraperitoneal injection of LPS in WT and AAV-shTRIM45 mice. BV2 cells were treated with LPS/ATP in vitro. Cognitive function was assessed by the Morris water maze. Nissl staining was used to evaluate histological and structural lesions. ELISA was used to dectect neuroinflammation. qPCR was used to detect the mRNA levels of inflammatory cytokines, NLRP3, and autophagy genes. Western blotting and immunofluorescence analysis were used to analyse the expression of the proteins. Changes in reactive oxygen species (ROS) in cells were observed by flow cytometry. Changes in mitochondrial membrane potential in BV2 cells were detected by JC-1 staining. Peripheral blood mononuclear cells were extracted from blood by density gradient centrifugation and then used for qPCR, western blotting and flow detection. To further explore the mechanism, we used the overexpression plasmids TRIM45 and Atg5 as well as siRNA-TRIM45 and siRNA-Atg5 to analyse the downstream pathway of NLRP3. The protein and mRNA levels of TRIM45 in peripheral blood mononuclear cells from sepsis patients were examined.

**Results:**

Knocking down TRIM45 protected against neuronal damage and cognitive impairment in septic mice. TRIM45 knockdown inhibited microglial pyroptosis and the secretion of inflammatory cytokines in vivo and in vitro, which was mediated by NLRP3/Gsdmd-N activation. Overexpression of TRIM45 could activate NLRP3 and downstream proteins. Further examination showed that TRIM45 regulated the activation of NLRP3 by altering Atg5 and regulating autophagic flux. It was also found that overexpression and knockdown of TRIM45 affected the changes in ROS and mitochondrial membrane potential. Thus, knocking down TRIM45 could reduce microglial pyroptosis, the secretion of proinflammatory cytokines, and neuronal damage and improve cognitive function. In addition, the level of TRIM45 protein in septic patients was increased. There was a positive linear correlation between APACHE II score and TRIM45, between SOFA score and TRIM45. Compared to group GCS > 9, level of TRIM45 were increased in group GCS ≤ 8.

**Conclusion:**

TRIM45 plays a key role in neuroinflammation caused by LPS, and the mechanism may involve TRIM45-mediated exacerbation of microglial pyroptosis via the Atg5/NLRP3 axis.

**Graphical Abstract:**

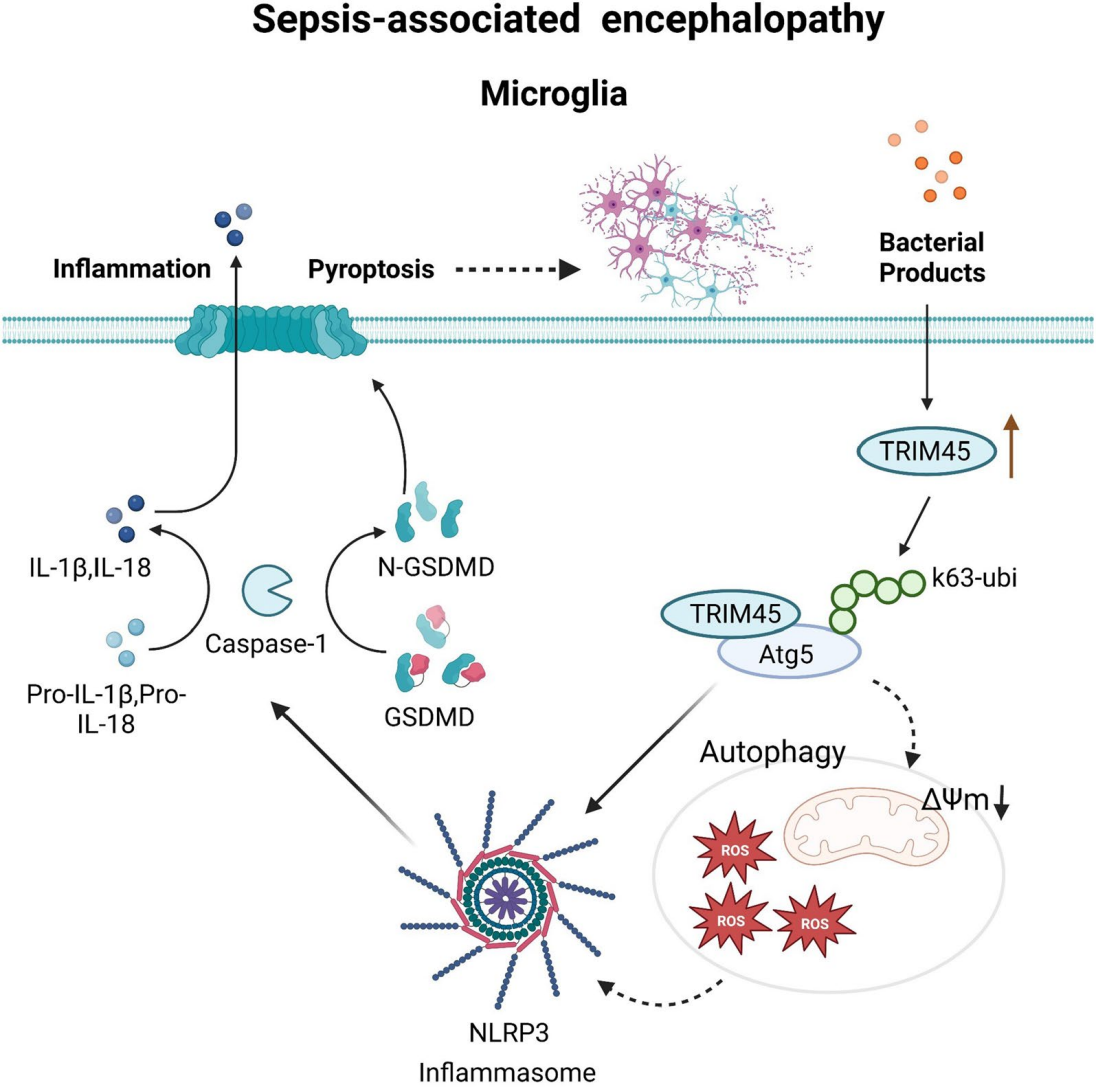

**Supplementary Information:**

The online version contains supplementary material available at 10.1186/s12974-023-02959-8.

## Introduction

SAE is a common and severe neurological complication of sepsis that mainly manifests as long-term cognitive impairment and mental illness and is closely related to increases in morbidity and mortality [1, 2]. Approximately 70% of patients with severe infection develop cognitive impairment after rehabilitation [3]. Despite the tremendous efforts of researchers, the pathological mechanism of SAE is still not fully understood. Therefore, studying the pathogenesis of SAE and finding potential targets to improve the quality of life and survival rate of SAE patients have scientific importance and clinical value [4, 5].

Many studies have reported that excessive neuroinflammation induced by sepsis-induced microglial activation in the brain is an important factor in cognitive impairment. Microglias are central innate immune cells and the main source of inflammatory mediators in the brain. Therefore, these cells are the focus of studies on neuroinflammation. Microglia is rapidly activated in response to various stimuli, including infectious and pathological stimuli or Aβ peptides. Activated microglias secrete many proinflammatory cytokines, such as TNF-α, IL-6 and IL-1β. Significantly increasing in neuroinflammation exacerbates neuronal damage and death, which leads to the behavioural and psychological symptoms of SAE [6, 7].

Activation of the NLRP3 inflammasome and the occurrence of pyroptosis in microglia are related to the pathogenesis of SAE [8, 9]. The NLRP3 inflammasome is the most well-defined inflammasome and is a multiprotein complex. The NLRP3 inflammasome is composed of Nod-like receptor (NLR), apoptosis-related spot-like protein containing caspase recruitment domain (ASC) and the caspase-1 precursor. NLR recognizes multiple stimuli, which forms complexes and cleaves pre-caspase-1 into activated caspase-1. Activated caspase-1 then cleaves the pore-forming protein gasdermin D (Gsdmd) before cleaving pre-IL-1 β and pre-IL-18, resulting in pyroptosis and IL-1 β and IL-18 secretion [10, 11]. Pyroptosis is closely related to SAE. Erbin protects against sepsis-associated encephalopathy by attenuating microglial pyroptosis via the IRE1α/Xbp1s-Ca^2+^ axis [12], and NLRP3/Caspase-1 pathway-induced pyroptosis mediates cognitive deficits in a mouse model of sepsis-associated encephalopathy [13]. However, the mechanism of NLRP3-mediated pyroptosis in SAE is still unclear.

Previous studies have shown that the motif (TRIM) protein family characterized by a ring finger, B-box zinc finger and spiral coil domain with conventional ubiquitin E3 ligase activity plays an indispensable role in regulating the inflammatory response, innate immunity, cell proliferation and apoptosis [14]. As a member of the TRIM family, TRIM45 stabilizes p53 through ubiquitin linked to K63 and is a brain tumour suppressor through its E3 ligase activity [15]. TRIM45 activates the NF-κB pathway through ubiquitin-induced TAB2 during cerebral ischaemia and reperfusion injury, which exacerbates microglia-mediated neuroinflammation and causes neuronal injury [16]. TRIM45 is highly expressed in human adult and embryonic brains [17]. However, whether and how TRIM45 plays a role in SAE remain unknown.

In this study, we found that the expression of TRIM45 was increased in septic encephalopathy and confirmed the colocalization of microglia and TRIM45 and that TRIM45 could regulate microglial pyroptosis and neuroinflammation in the brain. In summary, these findings showed that silencing TRIM45 could protect brain function from SAE-related damage and revealed the underlying mechanisms of TRIM45 in SAE.

## Materials and methods

### Animals

Male C57BL/6 mice (6–8 weeks old) were obtained from Beijing Vital River Laboratory Animal Technology (China). The mice were placed in controlled environments (12-h light/dark cycle; 22 °C; 50–60% humidity) and had free access to bacteria-free water and food. All animal housing and experiments were conducted in accordance with the ethical guidelines formulated by the Animal Experimental Committee of the First Affiliated Hospital of Wenzhou Medical University.

### Sepsis model

The sepsis mouse model was established by intraperitoneal injections of LPS (10 mg/kg; from *Escherichia coli* 055:B5, L2880, Sigma-Aldrich), and mice in the Sham group received intraperitoneal injections of an equal volume of PBS. Twenty-four hours later, the mice were anaesthetized and perfused with normal saline until the lungs were whitened. The hippocampus were collected for histological analysis or frozen in liquid nitrogen for rapid cryopreservation.

### Behavioural tests

#### Morris water maze (MWM) test

Ten days after the intraperitoneal injection of LPS, the MWM test was performed to evaluate the spatial learning and memory abilities of the mice. First, the mice were trained for 4 consecutive days, and then the experiment was carried out on the fifth day. The MWM consisted of a round steel pool (1.2 m in diameter and 0.6 m in height) with a hidden platform (0.1 m in diameter). The water in the pool was maintained at 23 °C, and the hidden platform was located in the southwest quadrant of the pool, approximately 1 cm under the surface. Propylene dye was used to make the water opaque. During the training period, each mouse was randomly placed in a different quadrant each day. The mice were allowed to find the platform within 60 s, and the time to reach the platform was recorded. If the platform was not found within 60 s, to the animal was placed on the platform to rest for 10 s. On the fifth day, the platform was removed, and the mouse was placed in the water in the quadrant opposite the platform. Each mouse swam freely for 60 s, and the number of passes and time spent in the target quadrant were recorded.

### Nissl staining

Nissl staining was performed to evaluate neuronal damage and loss. After paraffin embedding and sectioning (4 μm), brain tissues were stained with a 1% toluidine blue solution (Beyotime, C0117, China).

### Immunocytochemistry

BV2 cells were treated, soaked in 4% paraformaldehyde for 30 min and then permeabilized for 5 min with 0.2% Triton X 100. After being sealed with 5% BSA for 1 h, the cells were incubated with the indicated antibodies at 4 ℃ overnight. After being washed with PBS three times, the cells were incubated with Daylight 488-coupled secondary antibodies (1:500, CL488-10,594, Proteintech) or 594-coupled secondary antibodies (1:500, CL594-10594, Proteintech) for 1 h, and the nucleus was stained with DAPI. Images were recorded with a Leica confocal microscope.

Frozen embedded brain tissue sections were prepared. After the tissue sections were blocked with 5% bovine serum albumin for 1 h, 4-μm-thick brain slices were prepared and treated with rabbit anti-Gsdmd (1:100, A10164, ABclonal) or mouse anti-NLRP3 (1:100, AG-20B-0042, AdipoGen) overnight. Then, the sections were incubated with Daylight 488 -coupled secondary antibodies (1:500, CL488-10594, Proteintech) or 594-coupled secondary antibodies (1:500, CL594-10594, Proteintech) at room temperature for 1 h. After the sections were washed with PBS 3 times, the nuclei were stained with DAPI. Images were recorded with a Leica confocal microscope.

### Cell culture and treatments

The mouse microglia BV2 line, mouse astrocytes MA line and mouse hippocampal neuronal HT22 cell line were purchased from the Cell Bank of the Chinese Academy of Sciences (Shanghai, China) and cultured in DMEM (Gibco, USA) supplemented with 10% FBS and 1% penicillin/streptomycin at 37 °C in a humidified atmosphere containing 5% CO_2_. Lipopolysaccharide (LPS, L2880, Sigma‒Aldrich) and adenosine triphosphate (ATP, A3377, Sigma‒Aldrich) were used to activate the NLRP3 inflammasome in the cell model. For NLRP3 inflammasome activation, BV2 cells were treated with LPS (1 µg/mL) for 6 h and then treated with ATP (5 mM) for 30 min. DMSO was used as a vehicle control for the treatment conditions.

### Plasmids and siRNA transfection

Small interfering RNAs against TRIM45 were purchased from Hanbio Biotechnology (Shanghai, China). Small interfering RNAs against Atg5 (sc-41446, Santa Cruz) were purchased from Santa Cruz Biotechnology. Flag-Atg5, HA-TRIM45, HA-Ub, HA-Ub-K48 and HA-Ub-K63 were purchased from Limibio Biotechnology. These agents were transfected into BV2 cells using Lipofectamine RNAiMAX transfection reagent (13778075, Thermo Fisher Scientific) or Lipofectamine 2000 transfection reagent (11668500, Thermo Fisher Scientific). After 48 h, the cells were used for further experiments. The following sequences were used: siTRIM45 sense: 5′-GGTGGAGTGAAGGCTTTAACG-3′ and negative control siRNA (siNC) sense: 5′- UUCUCCGAACGUGUCACGUTT-3′.

### Determination of ROS by flow cytometry

ROS were examined by an ROS assay kit (Beyotime, S0033S, China). A total of 1 × 10^6^ BV2 cells were plated in 6-well plates and treated with LPS (1 μg/mL) for 6 h and ATP (5 mM) for 30 min. Then, the cell culture fluid was discarded, and the cells were carefully washed three times with DMEM. The cells were incubated with DMEM containing 10 µM dichlorodihydrofluorescein diacetate (DCFH-DA) for 30 min at 37 °C. Then, the cells were washed with DMEM three times to eliminate the excess DCFH-DA and collected in centrifuge tubes. Finally, the DCF fluorescence intensity was measured by flow cytometry at wavelengths of 485 nm and 535 nm. ROS levels were analysed by FlowJo Software.

### Detection of apoptosis in HT22 and BV2 cells by flow cytometry

Apoptosis in HT22 and BV2 cells were tested by Annexin V-FITC/PI Apoptosis Kit (AP101, MULTI SCIENCES). Adherent cells were collected by 0.25% EDTA digestion and centrifugation (4 °C, 1000 g, for 3–5 min), and 10^5^–10^6^ cells were collected and centrifuged (4 °C, 1000 g, for 3–5 min). Cell pellets were resuspended with 0.8–1 mL of cell staining buffer. Then, 5 μL of Annexin V-FITC staining solution was added, and 10 μL of PI staining solution was added. The mixture was mixed well and incubated at 37 °C for 5 min. Red fluorescence and blue fluorescence were detected by flow cytometry.

### JC-1 staining

The mitochondrial membrane potential in BV2 cells was determined via a JC-1 fluorescent probe (Beyotime, C2003S, China), and these cells were incubated with JC-1 working solution for 20 min at 37 °C. After being treated with LPS (1 μg/mL) for 6 h, the cells were treated with ATP (5 mM) for 30 min. Then, JC-1 buffer solution was used to wash the cells at least three times. The results were determined by calculating the rate of the green/red fluorescence intensity, which represented the level of mitochondrial disruption.

### PBMC collection, cytokine detection, antibodies, cell staining, and flow cytometry

Mouse eyeball blood (PBMC) was collected after isoflurane anaesthesia. Leukocytes were isolated from whole blood with red blood cell lysis buffer, and the remaining cells were stained. The antibodies and reagents used for flow cytometry are listed in Resources Table 1. Surface staining was performed in PBS containing 2% BSA or FBS (w/v). To detect cytokine production (IL-6 and TNF-α), lymphocytes were stimulated for 5 h in the presence of Cell Stimulation Cocktail (plus protein transport) (00-4975-93, Thermo Fisher). Intracellular cytokine staining (ICS) of IL-6 and TNF-α was performed with the Cytofix/Cytoperm Fixation/Permeabilization Kit (554714, BD Biosciences). Flow cytometry data were acquired with a BD Fortessa (BD Biosciences) and analysed using FlowJo (Tree Star).

**Table 1:** Flow antibody catalogue

Antibody	Source	Identifier
Fixable viability stain 700	BD	564997
Anti-Mouse CD45 APC-cy7	BD	557659
Anti-Mouse CD11b AF488	BD	557672
Anti-Mouse CD86 PE-cy7	BD	560582
Anti-Mouse F4/80 APC	Biolegend	123116
Anti-Mouse TNF-α PE	Biolegend	506306
Anti-Mouse IL-6 PE	BD	5204807

### TRIM45 adeno-associated virus infection

To downregulate the expression of TRIM45 in the mouse brain, we used an AAV9 vector carrying shRNA targeting mouse TRIM45 mRNA (Shanghai Genechem Co., Ltd.) and the core sequence of AAV-shTRIM45 was 5'-GGTGGAGTGAAGGCTTTAACG-3'. Stereotactic surgery to transfer the AAV vector was performed on male mice aged 11–12 weeks (25–30 g) that were anaesthetized with 350 mg/kg 4% chloral hydrate (i.p.). The mice were fixed on a stereotactic instrument, the skull was pierced by a drill, and the microsyringe was driven by a stepper motor. A total of 500 nL (2.5 × 10^12^ vg/mL) of virus solution was injected into the hippocampus. The speed was 50 nL/min. Two weeks later, the model was established in transfected mice.

### Reverse transcription real-time quantitative polymerase chain reaction (qRT-PCR)

Total RNA was extracted from BV2 cells with TRIzol reagent (Invitrogen, USA). RNA was reverse-transcribed to complementary DNA using RT-qPCR Fast Master Mix (Vazyme, China). Real-time fluorescence quantitative PCR was performed according to the manufacturer’s instructions. β-Actin and gapdh were used as internal controls for IL-1β, IL-18, TRIM45, Atg5, P62 and beclin1 mRNA expression analysis. Gene expression was quantified using the 2−ΔΔCt method. The gene primer sequences are listed in Table 2.

**Table 2:** Base sequence of each gene

qRT-PCR	5′ to 3′
Mouse	
TRIM45-F	TCAGGCAAGACTCATTGTCCT
TRIM45-R	ACGGATGTCCACTACTGAGAAT
Atg5-F	TGTGCTTCGAGATGTGTGGTT
Atg5-R	GTCAAATAGCTGACTCTTGGCAA
IL-18-F	GACTCTTGCGTCAACTTCAAGG
IL-18-R	CAGGCTGTCTTTTGTCAACGA
IL-1β-F	GCAACTGTTCCTGAACTCAACT
IL-1β-R	ATCTTTTGGGGTCCGTCAACT
P62-F	ATGTGGAACATGGAGGGAAGA
P62-R	GGAGTTCACCTGTAGATGGGT
beclin1-F	ATGGAGGGGTCTAAGGCGTC
beclin1-R	TCCTCTCCTGAGTTAGCCTCT
Huamn	
TRIM45-F	ACAAGCTCTGAGGGGTCAATA
TRIM45-R	CCACCTGAGCATCACATACAG
gapdh-F	GGAGCGAGATCCCTCCAAAAT
gapdh-R	GGCTGTTGTCATACTTCTCATGG

### ELISA

The levels of IL-1β and IL-18 in medium of BV2 cells and peripheral blood of mice were determined using an ELISA kit (MEIMIAN) according to the manufacturer’s instructions. The absorbance of the samples at a wavelength of 450 nm was measured with a BioTek microplate reader.

### Western blot analysis

RIPA lysis buffer was used to extract protein from hippocampal tissue or cultured cells, and a BCA protein detection kit was used to measure the concentration. Approximately 25 mg of protein was boiled for 5 min at 100 °C, separated by 10% SDS-PAGE, and transferred to PVDF membranes (1620177, Bio-Rad) The PVDF membranes were then blocked with 5% skim milk for 2 h at room temperature and incubated with primary antibodies (TRIM45 (ab169036, Abcam), NLRP3 (ab263899, Abcam), Gsdmd (ab219800, Abcam), IL-1β (ab16288, ABclonal), Atg5 (ET1611-38, HuaBio), LC3 (#4108, CST), SQSTM1/p62 (#23214, CST), beclin1 (JE59-31, Huabio), β-actin (HRP-60008, Proteintech), HA (51064-2-AP**,** Proteintech), Flag (80010-1-RR**,** Proteintech), Caspase3 (Abmart, T40044), cl-Caspase3 (Affinity, Asp175)) overnight at 4 °C. Then, the membranes were washed three times with TBST and then incubated with the appropriate horseradish peroxidase-labelled secondary antibodies. An enhanced chemiluminescence reagent was used to view the reaction. We measured the signal intensity using ImageJ AQ7. Standardization was performed using β-actin.

### Coimmunoprecipitation

The supernatants were incubated with Atg5 primary antibodies overnight at 4 °C, followed by the addition of 30 µL of protein A/G PLUS-agarose. After that, the solutions were incubated at 4 °C for 6–8 h. Then, the protein A/G PLUS-agarose was washed three times, and the protein attached to the agarose and linked to keap1 was extracted. We discarded the supernatant and resuspended the pellet in 45 µL of 2 × PAGE loading buffer, and boiled it for 5 min at 100 °C.

### Human sepsis specimens

To observe cell activity in the peripheral blood of normal subjects and patients with septicaemia, all septic patients hospitalized in the ICU from January 2023 to June 2023 were selected. Sepsis was determined according to the third internationally recognized definition of sepsis and septic shock, and the patients were minors, pregnant or had type 1 diabetes, aplastic diseases or immunosuppressive diseases or patients receiving immunosuppressive therapy were outside the scope of our study. Ethical review of human studies (KU2022-126) was performed by the Ethics Committee of the First Affiliated Hospital of Wenzhou Medical University, and the study was performed in accordance with the Helsinki Declaration and federal policy to protect human subjects. Each participating patient provided informed consent. For further study, peripheral blood mononuclear cells were obtained from blood by density gradient centrifugation.

### Statistical analysis

GraphPad Prism 8.3.0 was used to analyse the data and construct the graphs. The data are expressed as the mean ± SEM. Experiments with only 2 groups were analysed by the unpaired two-tailed Student’s t test. Single-factor experiments with > 2 groups were analysed with one-way analysis of variance (ANOVA) with Dunnett’s post hoc test. *P* < 0.05 was considered statistically significant.

## Results

### TRIM45 expression is upregulated in BV2 cells and the hippocampus by LPS

**Fig. 1:**
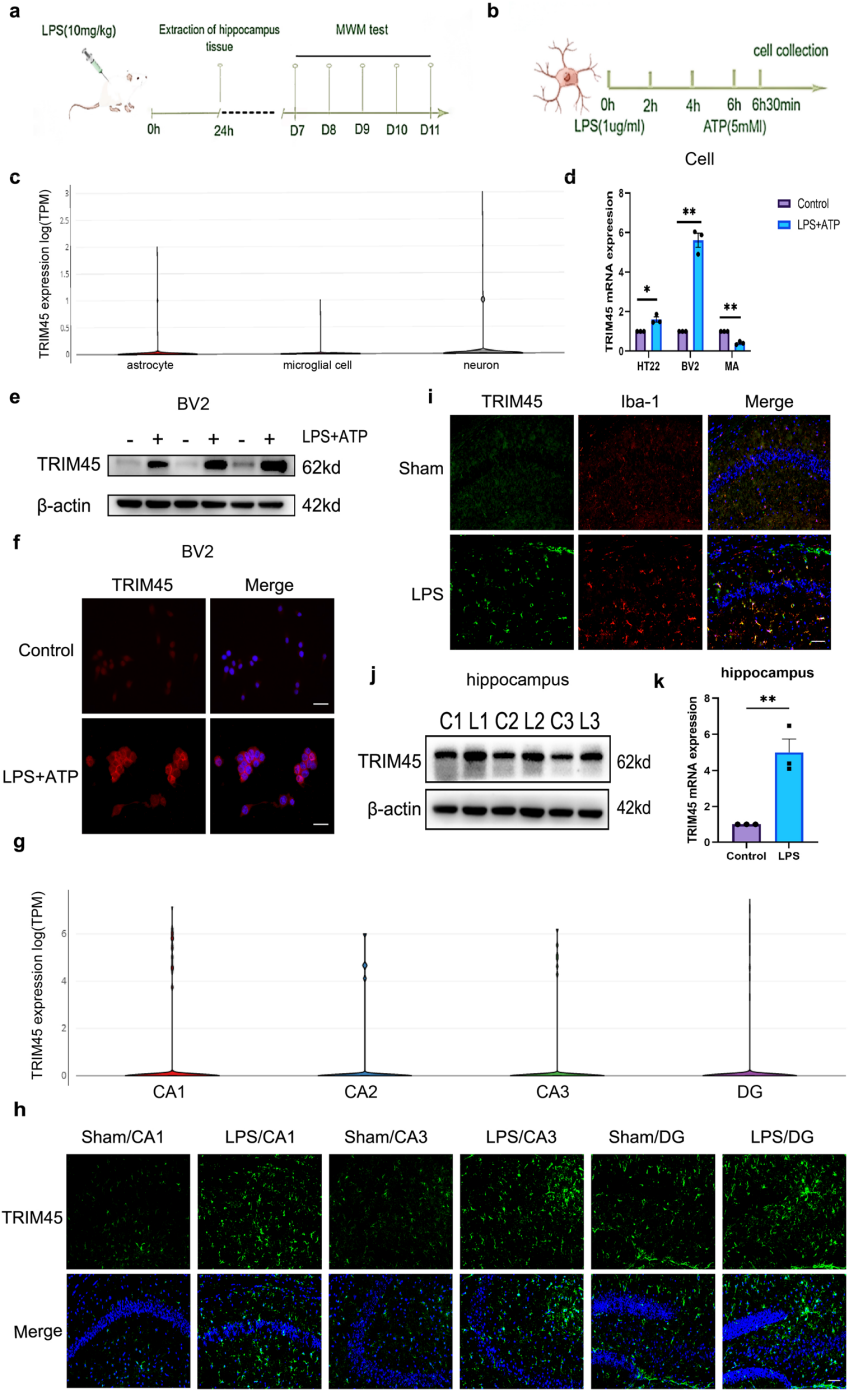
TRIM45 expression in BV2 cells and the hippocampus is upregulated by LPS.** a** Experimental design. Six- to eight-week-old male C57BL/6J mice were subjected to Sham treatment or intraperitoneal injection of LPS. The hippocampus was extracted at 24 h. MWM tests were carried out from the 7th to the 11th day. **b** BV2 cells were treated with LPS (1 µg/mL) for 6 h and then treated with ATP (5 mM) for 30 min. The cells and culture medium were collected for testing. **c** Distribution and analysis of TRIM45 in the mouse whole-brain atlas. **d** qRT-PCR analysis of TRIM45 expression in cells induced by LPS + ATP. BV2 (mouse microglia), MA (mouse astrocytes), HT22 (mouse hippocampal neurons) cells; *n* = 3 per group. **e** The levels of TRIM45 in BV2 cells were measured by western blotting; *n* = 3 per group. **f** Immunofluorescence analysis of TRIM45 expression in BV2 cells. Scale bars, 25 µm; *n* = 4 per group. **g** Distribution and scale of TRIM45 in the hippocampal tissue of mice. **h** Immunofluorescence analysis of TRIM45 expression in different regions of the hippocampus; scale bars, 50 µm. **i** Immunofluorescence analysis of TRIM45 expression in the hippocampal CA1 region of adult mice subjected to LPS for 24 h; scale bars, 50 µm. **j, k** TRIM45 expression in the hippocampus was analysed by western blotting and qRT-PCR. Data in **d, e, f** were analysed by unpaired t test. Data are presented as the mean ± S.E.M. from at least three independent experiments. *P < 0.05, **P < 0.01, ***P < 0.001 and ****P < 0.0001

To comprehensively evaluate the difference in TRIM45 in physiological conditions and septic encephalopathy, we used the single-cell website (https://singlecell.broadinstitute.org/single_cell) to examine the expression of TRIM45 in normal mouse brain cells (Fig. 1c). Specifically, we analysed three types of cells: neurons, microglia and astrocytes. In normal conditions, the expression of TRIM45 was highest in neurons, followed by astrocytes and microglia. To determine differences in TRIM45 between the septic encephalopathy and normal groups, we used LPS plus ATP to stimulate the HT22, BV2, and MA cells and found that TRIM45 was significantly increased in BV2 cells (Fig. 1d). In addition, compared with that in the control group, the expression of TRIM45 was increased in BV2 cells after modelling, as determined by western blotting and immunofluorescence analysis (Fig. 1e, f; The data analysis is shown in Additional file 1: Fig. S1). Abnormal hippocampus structure is associated with cognitive dysfunction [18, 19], and we again referenced the single-cell website to analyse the changes in TRIM45 in different regions of the hippocampus in normal mice. The results showed that TRIM45 was evenly distributed in the CA1, CA2, CA3 and DG regions (Fig. 1g). We examined changes in TRIM45 in different regions of the hippocampus in septic encephalopathy and found that the amount and intensity of TRIM45 fluorescence were increased in different regions of the hippocampus in septic mice compared to normal mice (Fig. 1h). In addition, we performed immunofluorescence colocalization and double staining of TRIM45 and Iba-1, and the results showed that TRIM45 colocalized with Iba-1 in the hippocampus of septic mice (Fig. 1i). Western blot and PCR showed that hippocampal expression of TRIM45 in septic mice was higher than that in normal mice (Fig. 1j, k; the western blotting data analysis is shown in Additional file 1: Fig. S2). These data show that the expression of TRIM45 was significantly upregulated in BV2 cells and the hippocampus after septic encephalopathy.

### TRIM45 promotes NLRP3 signalling activation in BV2 cells induced by LPS + ATP

**Fig. 2:**
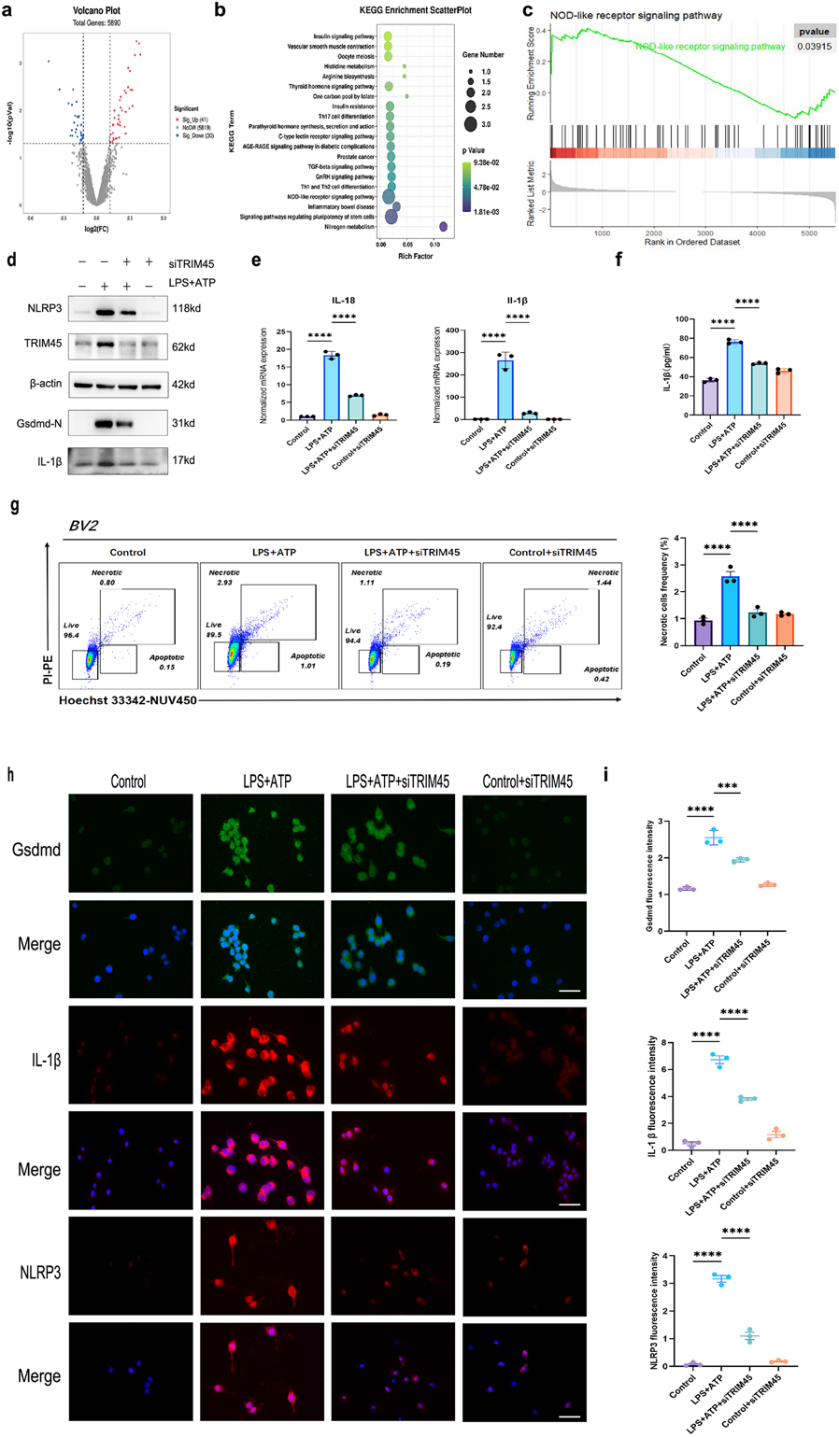
TRIM45 promotes NLRP3 signalling activation in BV2 cells induced by LPS + ATP.** a** Volcano graph of the control group and transfected group. The blue dots represent low expression, while the red dots represent high expression. **b** Pathway analysis of the enriched DEGs in GSE76328. **c** Gene set enrichment analysis of GSE76328**. d** Western blot analysis of pyroptosis-related proteins and TRIM45 in BV2 cells. **e** qRT-PCR analysis of IL-1β and IL-18 mRNA levels in BV2 cells; *n* = 3 per group. **f** BV2 cell secretion of IL-1β was determined by ELISAs; *n* = 3 per group. **g** Flow cytometry was used to analyse BV2 pyroptosis; *n* = 3 per group. **h, i** Immunofluorescence analysis of NLRP3, Gsdmd, and IL-1β levels in BV2 cells. Scale bars, 25 μm; *n* = 3 per group. Data in **e–i** were analysed by one-way ANOVA followed by Dunnett’s post hoc test. Data are presented as the mean ± S.E.M. from three independent experiments. *P < 0.05, **P < 0.01, ***P < 0.001 and ****P < 0.0001

To explore which signalling pathways were affected by TRIM45, we selected the GSE76328 dataset from the GEO database, which contained the mRNA expression of each gene in U251 cells (Human glioma cells) in the lv-TRIM45 transfection and negative control groups. After dividing the GSE76328 data into the normal control group and the viral transfection group, difference analysis was performed with the Limma package (parameters: *P* value < 0.05 and |log2FC|> 0.25), and 71 DEGs were obtained. Among these genes, 30 genes had low expression, and 41 genes had high expression. The results are visualized as a volcano map (Fig. 2a). We first analysed the 71 DEGs of GSE65682 by KEGG, and the Pathview package identified the NOD-like receptor signalling pathway. To avoid the one-sidedness caused by using only cross-point gene enrichment, we also performed GSEA on all genes in GSE76328. The reference gene set was c2.cp.kegg.v7.4.symbols.gmt (parameter: *P* value < 0.05), and the NOD-like receptor signalling pathway was shown to be expressed by GSEA (Fig. 2b, c). NLRP3, which is a member of the NOD-like receptor (NLR) family, contains a central nucleotide binding and oligomerization (NACHT) domain, which promotes self-oligomerization and has ATPase activity [20]. There is a close relationship between the NLR family and pyroptosis [21]. NLRP3-mediated pyroptosis is one of the pathogenic mechanisms of SAE. Based on the database and literature analysis, we transfected BV2 cells with siTRIM45 and studied the relationship between TRIM45 and pyroptosis. First, we detected the mRNA levels of IL-1β and IL-18 in cells. In response to LPS + ATP stimulation, TRIM45 knockout significantly decreased the levels of these inflammatory factors compared to LPS + ATP group (Fig. 2e). In addition, we used an ELISA kit to measure the level of IL-1β in extracellular fluid and found that knocking out TRIM45 decreased the production of IL-1β (Fig. 2f). Then, we used western blotting to measure the levels of pyroptosis-related proteins in the different groups. We were surprised to find that knocking down TRIM45 in the presence of LPS + ATP stimulation decreased the expression of NLRP3 and downstream proteins (Fig. 2d). These results were confirmed by immunofluorescence analysis (Fig. 2h, i). Thus, we hypothesize that the degradation of NLRP3 may occur via autophagy rather than proteasomal degradation. In addition, we used a Hoechst 33,342/PI double staining kit to detect necrosis by flow cytometry and found that knocking out TRIM45 could decrease necrosis caused by pyroptosis (Fig. 2g). In summary, these results suggest that TRIM45 regulates the NLRP3-mediated pyroptosis pathway in BV2 cells.

### TRIM45 regulates autophagy proteins associated with NLRP3

**Fig. 3:**
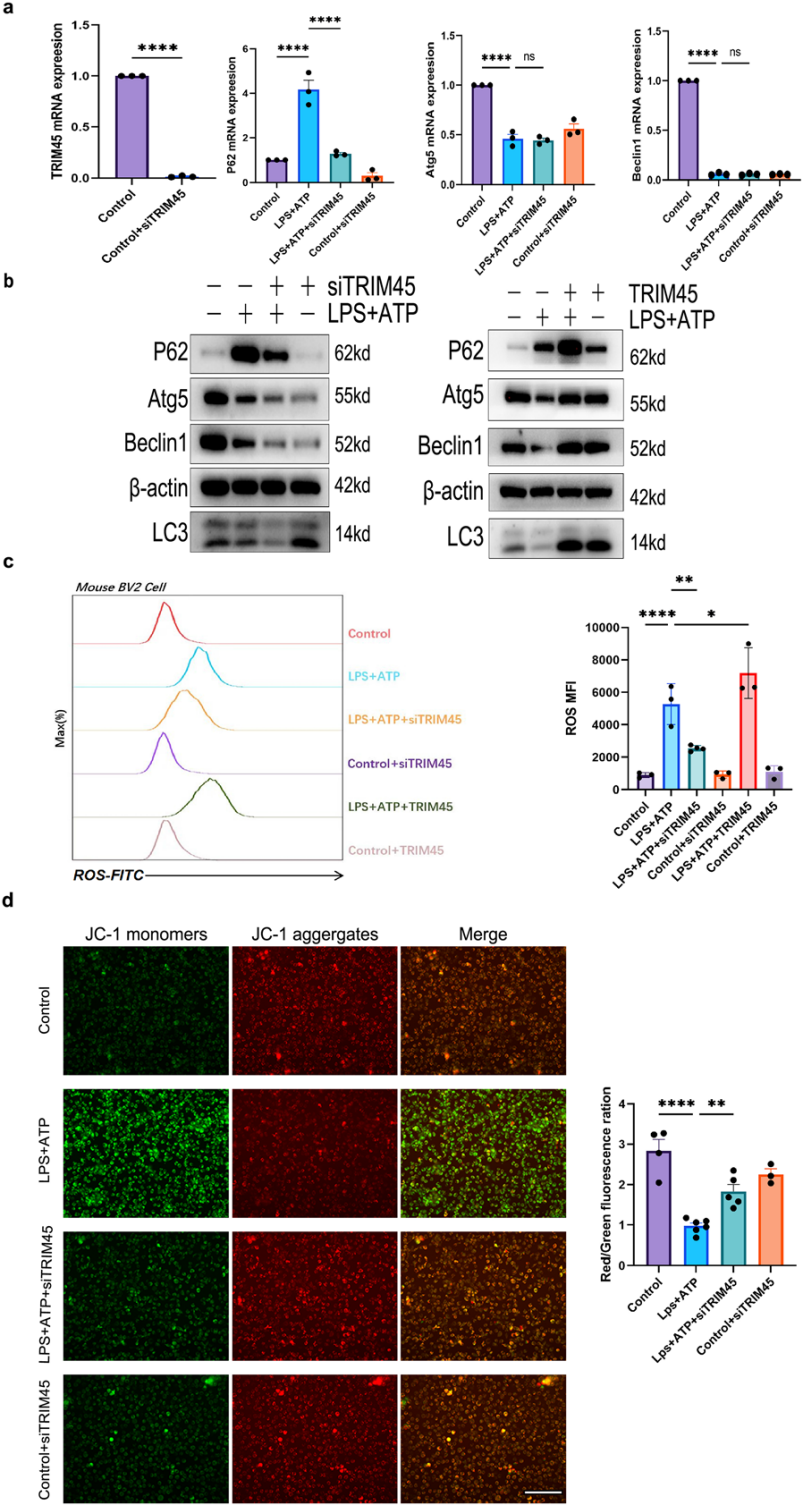
TRIM45 regulates autophagy proteins associated with NLRP3.** a** qRT-PCR analysis of p62, Atg5, and beclin1 mRNA levels in BV2 cells; *n* = 3 per group. **b** The autophagy proteins p62, beclin1, Atg5 and LC3 in BV2 cells were measured by western blotting.** c** Changes in ROS in BV2 cells in the different groups; *n* = 3 per group. **d** JC-1 staining to detect mitochondrial membrane potential and cellular morphology was examined by a phase-contrast microscope. (scale bar = 75 μm); *n * ≥ 3 per group. Data in **a, c, d** were analysed by one-way ANOVA followed by Dunnett’s post hoc test. Data are presented as the mean ± S.E.M. from three independent experiments. *P < 0.05, **P < 0.01, ***P < 0.001 and ****P < 0.0001

It has been reported that autophagy-related proteins such as p62, Atg5, and beclin1 play important roles in regulating the activation of NLRP3 inflammatory bodies [22–25]. We explored the relationship between TRIM45 and autophagy proteins. We exposed BV2 cells to siTRIM45 and measured the changes in p62, Atg5 and beclin1 mRNA in response to LPS + ATP stimulation. The results showed that in the model, knocking down TRIM45 decreased the mRNA level of p62, but there was no significant change in beclin1 or Atg5 (Fig. 3a). Similarly, in the model, knocking down TRIM45 decreased protein expression of p62, beclin1, Atg5 and LC3II/I, while overexpressing TRIM45 increased Atg5, p62, beclin1, and LC3II/I (Fig. 3b). We hypothesize that there may be degradation or posttranscriptional modification of Atg5 and beclin1. The imbalance in redox homeostasis under pathological conditions can lead to excessive production of ROS. Autophagy is the main cellular defence against oxidative stress or related conditions, leading to the accumulation of damaged proteins or organelles [26, 27]. The results showed that in response to LPS + ATP, knockout of TRIM45 could significantly reduce the production of reactive oxygen species, and ROS are involved in NLRP3 activation (Fig. 3c). Mitochondrial membrane potential damage is an important source of ROS production in cells [28]. We further studied the effect of altering TRIM45 on membrane potential. The results showed that in response to LPS + ATP stimulation, knockout of TRIM45 improved mitochondrial membrane potential and reduced mitochondrial membrane damage (Fig. 3d). According to these results, in response to LPS + ATP stimulation, TRIM45 intervention affected autophagy-related proteins, changed mitochondrial membrane potential and regulated ROS production.

### TRIM45 regulates Atg5 based on its E3 ubiquitin ligase activity

**Fig. 4:**
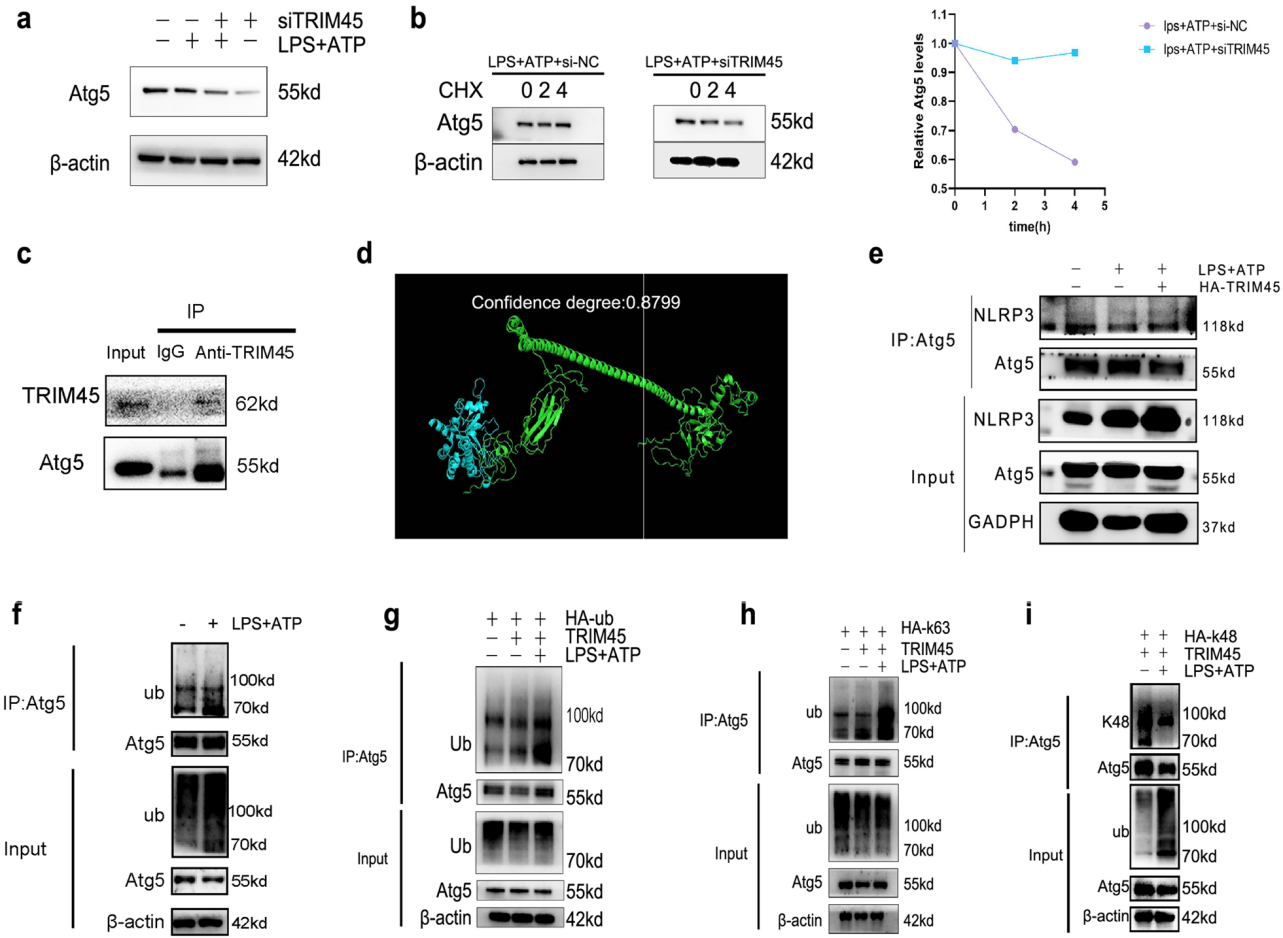
TRIM45 regulates Atg5 based on its E3 ubiquitin ligase activity.** a** Western blot analysis of Atg5 expression in BV2 cells in response to different interventions. **b** BV2 cells with TRIM45 knockdown or control cells were treated with cycloheximide (CHX) for 0, 2 and 4 h. **c** BV2 cells were immunoprecipitated with IgG and anti-Atg5 antibodies, and the expression of TRIM45 and Atg5 was detected by western blotting. **d** Protein confidence analysis of Atg5 and TRIM45 was performed by high-throughput interactive group (TRIM45 (green), Atg5 (blue)). **e** BV2 cells were transfected with HA-TRIM45 for 48 h. BV2 cells were immunoprecipitated with an anti-Atg5 antibody and then subjected to western blot analysis with an anti-NLRP3 antibody. **f** Co-IP analysis of Atg5 ubiquitination in BV2 cells. The cell extracts were collected for IP with anti-Atg5 beads, followed by IP analysis with the indicated antibodies. **g** HEK293T cells were transfected with TRIM45 and HA-tagged ubiquitin plasmids with or without LPS + ATP treatment. The cell extracts were collected for IP with anti-Atg5 beads, followed by IP analysis with the indicated antibodies. **h, i** HEK293T cells were transfected with plasmids TRIM45 and HA-tagged k63 or k48 ubiquitin with or without LPS + ATP treatment. The cell extracts were collected for IP with anti-Atg5 beads, followed by IP analysis with the indicated antibodies

Because TRIM45 is an E3 ubiquitin ligase, it functions as a ubiquitin substrate. Previous studies have shown that TRIM45 can affect the protein levels of Atg5 but does not change the mRNA level. Therefore, we examined whether TRIM45 affected the Atg5 protein through its E3 ligase activity. In response to LPS + ATP stimulation, the expression of Atg5 was decreased in TRIM45-knockdown cells (Fig. 4a). We used CHX to study the half-life of the Atg5 protein in the LPS + ATP + si-NC group and LPS + ATP + siTRIM45 group. The results showed that siTRIM45 accelerated the degradation of Atg5 (Fig. 4b). We examined the amino acid sequences of TRIM45 and Atg5 in the official NCBI website and analysed the confidence of the two proteins using high-throughput interaction omics. It was found that the score was greater than 0.7. There is a possibility of binding between these two proteins. Co-IP assays showed that TRIM45 could bind to Atg5 in BV2 cells (Fig. 4c, d). In addition, some studies have shown that USP22 can inhibit the NLRP3 inflammasome via Atg5-mediated autophagy, and Atg5 can directly bind to NLRP3. Therefore, we used coimmunoprecipitation to examine whether knocking down TRIM45 affected the binding of Atg5 and NLRP3. Compared to the control group, the LPS + ATP group and LPS + ATP + HA-TRIM45 group had reduced binding of Atg5 and NLRP3 (Fig. 4e). The next step was to explore whether TRIM45 can mediate ubiquitin modification of the Atg5 protein. We found that ubiquitin levels in the LPS + ATP group were increased compared to those in the control group (Fig. 4f). It has been reported that the ubiquitin chains of TRIM45 are mainly K63 and K48. K63 plays a role in stabilizing protein structure, while K48 promotes protein degradation [29, 30]. HEK293T cells were transfected with HA-ub, HA-k63ub or HA-k48ub and TRIM45. The results showed that TRIM45 promoted Atg5 ubiquitination through K63 but not K48 (Fig. 4g–i). These results show that TRIM45 stabilizes Atg5 by coupling Atg5 with the K63 ubiquitin chain.

### TRIM45 regulates the NLRP3 pathway in an Atg5-dependent manner

**Fig. 5:**
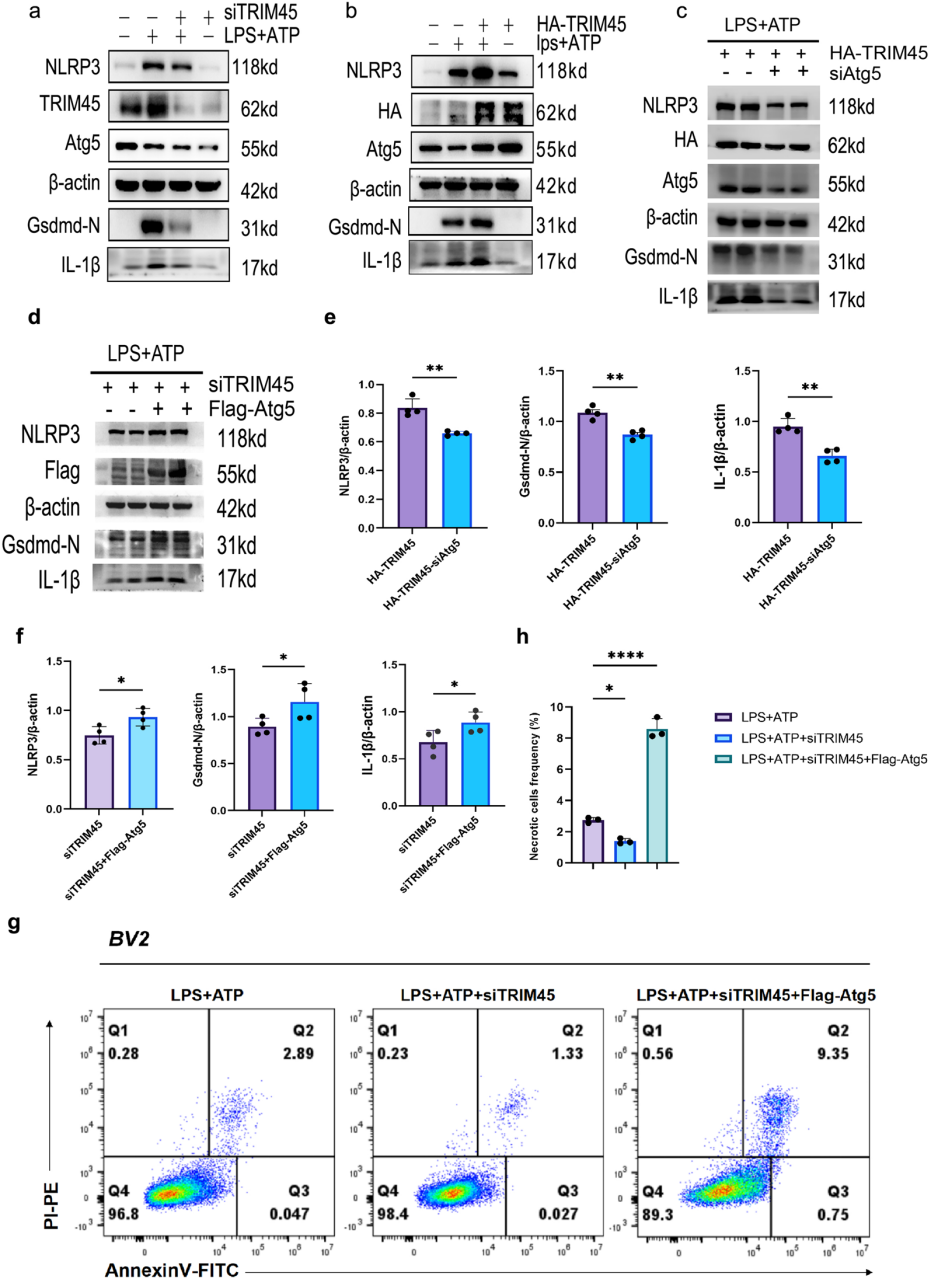
TRIM45 regulates the NLRP3 pathway in an Atg5-dependent manner.** a, b** Western blot analysis and quantification of NLRP3, Gsdmd-N, Atg5 and IL-1β levels in BV2 cells transfected with siTRIM45 or HA-TRIM45 and stimulated by LPS + ATP; *n* = 3 per group. **c** BV2 cells were infected with siTRIM45 and Flag-Atg5; *n* = 4 per group. **d** BV2 cells were infected with HA-TRIM45 and siAtg5; *n* = 4 per group. **e, f** Statistical analysis of Figures c and d. **g, h** Flow cytometry was used to analyse BV2 pyroptosis; *n* = 3 per group. Data in **a, b, h** were analysed by one-way ANOVA followed by Dunnett’s post hoc test. Data in **c, d** were analysed by unpaired t test. Data are presented as the mean ± S.E.M. from at least three independent experiments. *P < 0.05, **P < 0.01, ***P < 0.001 and ****P < 0.0001

It has been reported that Atg family proteins affect the NLRP3 pathway by regulating autophagy [31–33]. Previous studies have shown that TRIM45 intervention can change the Atg5 or NLRP3-mediated pyroptosis pathway; TRIM45 promotes the ubiquitination of Atg5 at K63. Therefore, we aimed to determine whether TRIM45 regulated the NLRP3 pathway in an Atg5-dependent manner. First, western blotting showed that upregulating TRIM45 expression increased the protein expression of Atg5 and NLRP3 in LPS + ATP-stimulated BV2 cells, and knocking down TRIM45 decreased the protein expression of Atg5 and NLRP3 (Fig. 5a, b; the statistical analysis is in Additional file 1: Fig. S3). More importantly, the effect of knocking down TRIM45 on inhibiting NLRP3 activation was reversed by the overexpression of Atg5 (Fig. 5d, f). And the effect of up-regulation of TRIM45 on promoting NLRP3 activity was reversed by the knockdown of ATG5 (Fig. 5c, e). In addition, we verified the pyroptosis level of BV2 cells in different groups. Compared with LPS + ATP group, knocking down TRIM45 improved the proportion of necrosis in Q2 region. On the contrary, knocking down TRIM45 and overexpressing Atg5 increased the proportion of necrosis in Q2 region (Fig. 5g, h, Q2 region represents late apoptosis or necrotic cells). In summary, these findings suggest that TRIM45 promotes NLRP3 activation in an Atg5-dependent manner.

### Inhibiting TRIM45 can reduce neuronal injury in SAE

**Fig. 6:**
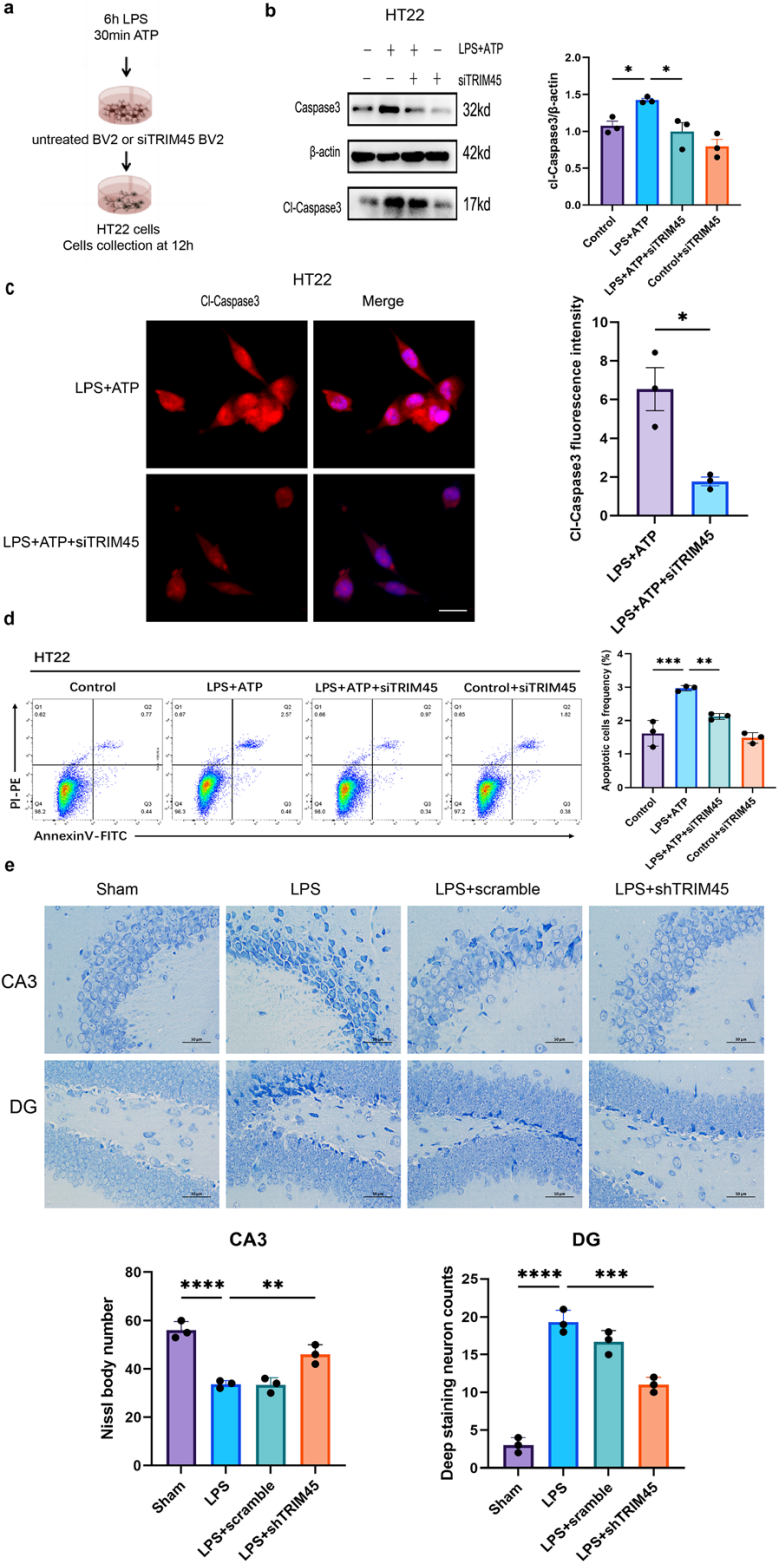
Inhibiting TRIM45 can reduce neuronal injury in SAE.** a** BV2 cells were treated with siTRIM45, cocultured with neurons in a transwell system and subjected to LPS + ATP. **b** Levels of Caspase-1 and Cl-Caspase-1 in HT22 cells were measured by western blotting; *n* = 3 per group. **c** Immunofluorescence analysis of Cl-Caspase-1 levels in HT22 cells. Scale bars, 20 um; *n* = 3 per group. **d** Apoptosis in HT22 cells was detected by flow cytometry; *n* = 3 per group. **e** Neuronal damage in the hippocampal region was assessed by Nissl staining. Scale bar, 50 µm; *n* = 3 per group. Data in **b, d, e** were analysed by one-way ANOVA followed by Dunnett’s post hoc test. Data in **c** were analysed by unpaired t test. Data are presented as the mean ± S.E.M. from three independent experiments. *P < 0.05, **P < 0.01, ***P < 0.001 and ****P < 0.0001

Previous experiments have shown that TRIM45 increased the expression of proinflammatory cytokines in BV2 cells induced by LPS + ATP. To further confirm whether TRIM45 knockout could directly protect neurons by regulating microglia in septic encephalopathy, we transfected BV2 cells with siTRIM45 and used a BV2-HT22 coculture system (Fig. 6a). Western blotting showed that knocking down TRIM45 downregulated Caspase3 and Cl-Caspase3 compared to the LPS + ATP group (Fig. 6b). Immunofluorescence showed that compared with LPS + ATP group, Cl-caspase3 fluorescence intensity decreased in TRIM45 knockdown group in HT22 cells (Fig. 6c). In addition, we used flow cytometry to analyse HT22 cells exposed to the different interventions and found that knockdown of TRIM45 could reduce apoptosis in HT22 cells (Fig. 6d). Nissl staining was performed to evaluate the neuropathy induced by LPS. As expected, neurons in the CA3 and DG regions of the hippocampus of LPS-induced mice showed extensive damage, nuclear pyknosis and cytoplasmic atrophy, while neuronal damage in the hippocampal CA3 and DG regions was decreased in shTRIM45 mice (Fig. 6e). These data suggest that TRIM45 inhibition in microglia decreases the neurotoxic effects in SAE.

### Knockdown of TRIM45 can inhibit pyroptosis and improve cognitive impairment and may be a target for treating patients with sepsis

**Fig. 7:**
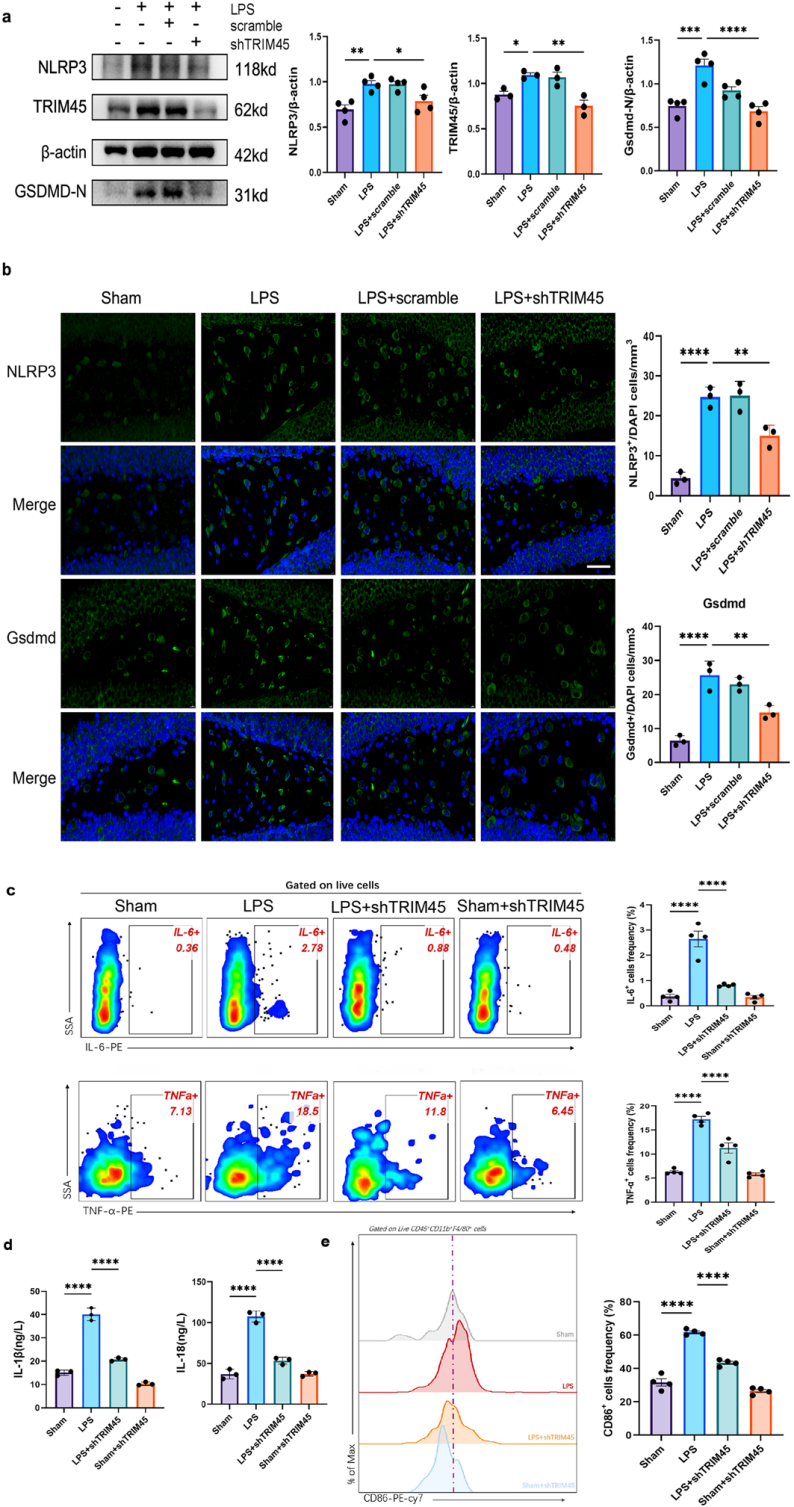

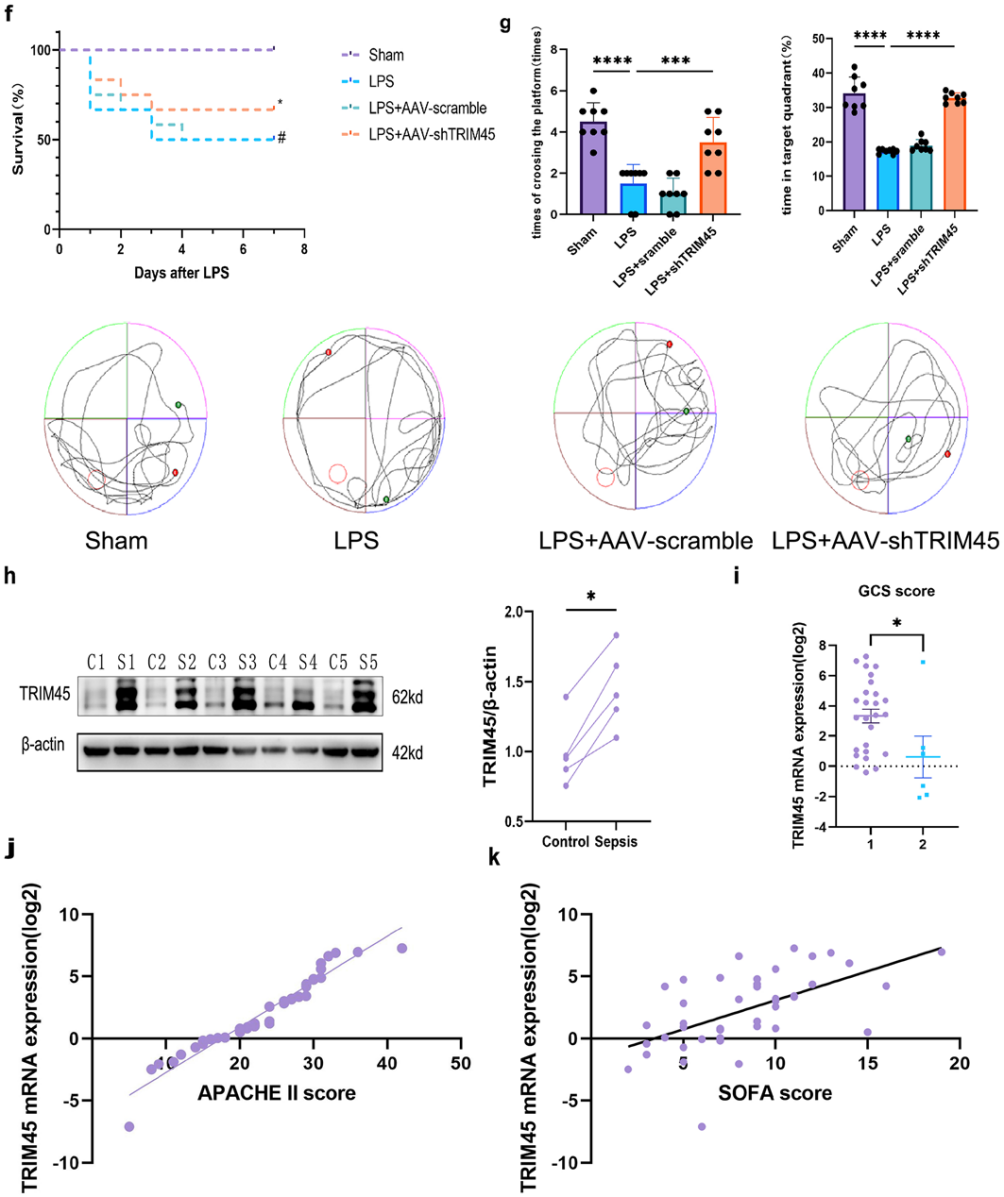
Knockdown of TRIM45 can improve cognitive impairment and may be a target for treating patients with sepsis.** a** The levels of NLRP3, Gsdmd-N and TRIM45 in the mouse hippocampus were measured by western blotting; *n* ≥ 3 per group. **b** The numbers of Gsdmd^+^/DAPI and NLRP3^+^/DAPI positive cells in the hippocampus were measured by immunofluorescence staining. Scale bar, 10 µm; *n* = 3 per group. **c** The levels of secreted IL-6 and TNF-α were determined by flow cytometry; *n* ≥ 3 per group. **d** The levels of secreted IL-1β and IL-18 were determined by ELISA; *n* = 3 per group. **e** Flow cytometric analysis of M1 (CD86^+^) mononuclear macrophages in the serum of different groups of mice; *n* = 3 per group. **f** 7-day survival rates of mice induced by LPS. *n* = 12 per group. #*P* < 0.05 Sham vs. LPS, *P < 0.05 LPS vs. LPS + AAV-TRIM45. **g** Morris experiment and corresponding statistical analysis; *n* ≥ 8 per group. **h** The levels of TRIM45 in peripheral blood monocytes were measured by western blotting (c: control, s: sepsis); *n* = 5 per group. **i** Relation between TRIM45 and the GCS score; Group 1: GCS score of patients ≤ 8, *n* = 26; Group 2: GCS score of patients > 9, *n* = 6. **J** Correlation between TRIM45 expression and Acute Physiologic and Chronic Health Evaluation II scores in septic patients (R^2^ = 0.9342, *P* < 0.0001); *n* = 44. **k** Correlation between TRIM45 expression and SOFA scores in septic patients (R^2^ = 0.3365, *P* < 0.0001); *n* = 44. Data in **f** were assessed by the Kaplan–Meier log-rank test. Data in **j, k** were analysed by Spearman correlation test. Data in **i** were analysed by unpaired t test, and others were analysed by one-way ANOVA followed by Dunnett’s post hoc test. Data are presented as the mean ± S.E.M. from at least three independent experiments. n.s. for *P* > 0.05, *P < 0.05, **P < 0.01, ***P < 0.001 and ****P < 0.0001

These experiments have proven that there is a relationship between TRIM45 and pyroptosis at the cellular level. To further explore the relationship between TRIM45 and pyroptosis in vivo, AAV-Scramble or AAV-shTRIM45 mice were stimulated with LPS and examined by immunoblotting and immunofluorescence. The results showed that TRIM45 knockdown inhibited NLRP3 and Gsdmd expression (Fig. 7a, b). We collected eyeball blood from different groups of mice. IL-1β and IL-18 were analysed by ELISA (Fig. 7d). IL-6 and TNF-α were analysed by flow cytometry (Fig. 7c). It was found that the levels of IL-1β, IL-18, IL-6 and TNF-α were decreased in the shTRIM45 group after LPS stimulation. After determining the relationship between TRIM45 and inflammatory factors, we examined the polarization of M1 macrophages in peripheral blood of mice. We used CD86 to label the cells, and the results showed that knocking down TRIM45 could reduce the proportion of M1 macrophages (Fig. 7e). In addition, we explored the effect of TRIM45 intervention on the 7-day survival rate of septic mice (Fig. 7f). Compared to Sham group, the survival rate in the LPS group was significantly decreased, and the 7-day survival rate was approximately 50% (6 of 12 mice survived). In addition, Compared to LPS group, the LPS + AAV-shTRIM45 group showed improved survival rate, and the survival rate was 66.7% (8 of 12 mice survived). Furthermore, we aimed to verify whether TRIM45 was related to cognitive impairment. We performed MWM tests to examine spatial learning and memory. The results showed that in response to LPS conditions, the animals that were pretreated with shTRIM45 took less time to reach the hidden platform, and the TRIM45-knockdown mice spent more time wandering in the targeting quadrant (Fig. 7g, Additional file 1: Fig. S4). Based on these results, we suggest that TRIM45 can be used as a regulatory protein or marker of sepsis. We screened 47 patients with sepsis, the details of which are shown in Table 3. We extracted peripheral monocytes from patients with sepsis and analysed TRIM45 by PCR and WB. The results showed that the levels of TRIM45 protein in the septic group were higher than those in the normal group (Fig. 7h). Since there are no exact diagnostic criteria for SAE, we found that the Glasgow Coma Score (GCS), Sequential Organ Failure Assessment Score (SOFA), and Acute Physiology and Chronic Health Evaluation (APACHE II) score may be associated with SAE, and so we examined the correlation between TRIM45 and these indicators [34–37]. We analysed TRIM45 mRNA levels in peripheral blood monocytes and the APACHE II score, SOFA score in some patients with sepsis by linear regression analysis, and compare the level of TRIM45 in the two groups (group1: GCS score ≤ 8; group2: GCS score > 9) for patients with sepsis. The results showed that there was a positive linear correlation between APACHE II score and TRIM45, between SOFA score and TRIM45 (Fig. 7j, k). Compare to group2, level of TRIM45 were increased in group1 (Fig. 7i). Therefore, reducing brain levels TRIM45 may alleviate the brain damage caused by sepsis, and TRIM45 is one of the targets of sepsis. Therefore, reducing brain levels TRIM45 may alleviate the brain damage caused by sepsis, and TRIM45 maybe one of the targets of sepsis.

**Table 3:** Baseline clinical characteristics of the study subjects

Patient group			
Characteristics	All patients	Survivors	Nonsurvivors
Demographics and underlying conditions			
Number of patients	47	36	11
Males, number (%)	37 (78.7%)	31 (86.1%)	6 (54.5%)
Age (years)	73 [60.25,81.75]	70 [59,79]	82 [65.5,88.5]
Cardiac functional insuffificiency, number (%)	10 (21.3%)	8 (22.2%)	2 (18.1%)
Renal functional insuffificiency, number (%)	14 (27.8%)	10 (27.8%)	4 (36.4%)
Hypertension, number (%)	27 (57.4%)	18 (50%)	9 (81.8%)
Diabetes mellitus, number (%)	24 (51.1%)	17 (47.2%)	7 (63.6%)
Disease severity, number (%)			
Septic shock	23 (48.9%)	14 (38.9%)	9 (81.8%)
Baseline parameters			
APACHE II score	23.11 ± 0.975	22.7 ± 1.165	24.46 ± 1.686
SOFA score	8.21 ± 0.515	8.23 ± 0.612	8.15 ± 0.946
Site of infection, number (%)			
Lung	21 (44.7%)	14 (38.9%)	7 (63.6%)
Abdomen	20 (42.6%)	16 (44.4%)	4 (36.3%)
Urinary tract	3 (6.4%)	3 (8.3%)	0
Other	3 (6.4%)	3 (8.3%)	0
Intervention, number (%)			
Mechanical ventilation	34 (723%)	23 (63.9%)	11 (100%)
Renal-replacement therapy	22 (46.8%)	16 (44.4%)	6 (54.5%)
Length of stay			
In the ICU (days)	23.5 [12,40.25]	20 [12, 33]	38 [16, 55]

Data are presented as median [interquartile range] or mean ± SEM or *n* (%)

## Discussion

TRIM45 functions as an E3 ubiquitin ligase and participates in a variety of cellular signalling pathways. TRIM45 plays an inhibitory role in brain tumours, and overexpression of TRIM45 can inhibit the proliferation and tumorigenicity of glioblastoma cells in vivo and in vitro. Further analysis showed that TRIM45 induced p53 polyubiquitination with a K63 linkage [15]. Other studies have shown that TRIM45 plays a role in ischaemic stroke. TRIM45 also uses K63 ubiquitin and TAB2 to control microglial NF-kB signalling. Microglia-specific knockdown of TRIM45 in mice can significantly reduce infarct size and neurological deficit scores [16]. Although TRIM45 is highly expressed in human and embryonic tissues, its role in the central nervous system of mice with septic encephalopathy has not been explored. In this study, we showed that TRIM45 regulated the TRIM45–Atg5–NLRP3 axis to regulate microglial pyroptosis in the central nervous system of mice with SAE.

SAE is a common and serious complication of sepsis with high morbidity and mortality. Some studies have shown that there is a link between brain damage and cognitive impairment in SAE [38, 39]. Consistent with previous studies, intraperitoneal injection of LPS resulted in severe learning and memory impairment mice, as shown by the water maze test, and a lack of TRIM45 alleviated cognitive impairment in septic mice. More importantly, we found that TRIM45 was upregulated in the hippocampus of septic mice compared to that of untreated mice. We hypothesized that this improvement was due to SAE. The hippocampus is the structure associated with cognitive impairment in sepsis, and impairment of the CA1, CA3, and DG regions is used in many studies to explore cognitive function [40, 41]. In the Transwell coculture system in vitro, the downregulation of TRIM45 expression inhibited microglial activation induced by LPS + ATP and reduced neuronal apoptosis. We observed severe neuronal damage in the CA1 and DG regions of mice in the LPS group, and AAV-shTRIM45 mice had reduced damage caused by neuronal inflammation in the brain. This study was the first to show the role of TRIM45 in SAE and that it is important in alleviating cognitive impairment induced by SAE.

During septic encephalopathy, neuroinflammation in the brain causes microglial activation, and the secretion of proinflammatory factors can damage surrounding brain tissue [42]. Microglia are the main sites of abnormal activation and pyroptosis mediated by NLRP3 inflammatory bodies. Once the NLRP3 inflammatory body is activated, its downstream active proteins Gsdmd-N-terminal and cl-caspase-1 are increased, resulting in pyroptosis and IL-1β and IL-18 secretion [43, 44]. We analysed the GSE76328 dataset and found that TRIM45-related differentially expressed genes were clustered with nod-like pathways. Then, we explored the relationship between TRIM45 and the NLRP3-mediated pyroptosis pathway and found that knockdown of TRIM45 downregulated pyroptosis proteins in BV2 cells stimulated with LPS + ATP. Overexpression of TRIM45 upregulated pyroptosis proteins. In vivo, pyroptosis-related proteins were decreased in AAV-shTRIM45-treated septic mice compared to septic mice. These results indicate that TRIM45 is related to pyroptosis and that knocking down TRIM45 can inhibit the pyroptosis pathway.

Autophagy is the main catabolic process in cells. The fate of damaged cells is determined by the rapid degradation of harmful factors and the coordination of survival and death processes [45, 46]. Autophagy dysfunction is associated with a variety of inflammation-related diseases [47, 48]. Studies have shown that the TRIM family can affect disease progression through autophagy. Knockout of TRIM65 can inhibit autophagy and cisplatin resistance in A549/DDP cells by regulating miR-138-5p/ATG7 [49]. TRIM22 inhibits osteosarcoma progression by destroying NRF2 and activating ROS/AMPK/mTOR/autophagy signalling [50]. TRIM39 deficiency can inhibit tumour progression and autophagy in colorectal cancer by inhibiting Rab7 activity [51]. However, the relationship between TRIM45 and autophagy is still unclear. We found that in the model, knocking down or overexpressing TRIM45 affected the protein levels of p62, beclin1, and Atg5, but at the transcriptional level, knocking down TRIM45 had no effect on beclin1 or Atg5. We hypothesize that there is a protein modification process. Autophagy is regulated by ATG proteins, and Atg5 is indispensable for the formation of autophagic vesicles [52]. Some studies have shown a relationship between Atg5 and NLRP3 inflammatory bodies [53]. In ulcerative colitis, EZH2 reduces colonic inflammation through the Atg5-NLRP3 axis [31]. In NLRP3 inflammasome-related diseases, USP22 inhibits NLRP3 inflammatory bodies through Atg5-dependent autophagy to degrade NLRP3 [23]. Therefore, we further explored the relationship between TRIM45 and Atg5.

TRIM45 has E3 ubiquitin ligase activity. Previous studies have shown that TRIM45 can modify downstream proteins via ubiquitin. We hypothesize that there is also an interaction between TRIM45 and Atg5. We found that there was binding between TRIM45 and Atg5. We further explored the type of ubiquitin modification of Atg5 mediated by TRIM45. The results show that TRIM45 modifies Atg5 with K63 ubiquitin but not K48.

After verifying the relationship between TRIM45 and Atg5, we focused on how Atg5 affects NLRP3. We overexpressed or knocked down TRIM45 and examined changes in NLRP3 and downstream proteins. The results showed that TRIM45 regulated NLRP3 in an Atg5-dependent manner. Considering the correlation between autophagy, ROS and mitochondrial membrane potential [54, 55], we also examined the changes in these indices in the different groups. The results showed that knocking down TRIM45 could improve mitochondrial membrane potential and reduce ROS production. However, these two indicators are the prerequisite for activating NLRP3. In addition, IP experiments showed that Atg5 could directly act on NLRP3. Based on these results, we believe that TRIM45 plays a role in autophagy, mitochondrial membrane potential and ROS.

However, there are some limitations in our study that cannot be ignored. We used BV2 cell line to replace primary microglia for in vitro experiments. Even though BV2 cell line has been widely used in neuroscience research, it can be used as an alternative model of primary microglia. In the future, we will continue to use primary microglia to conduct in-depth research and increase the reliability of the conclusions. Some studies have shown that TRIM50, which is a member of the TRIM family, can directly induce NLRP3 oligomerization to promote the activation of NLRP3 inflammatory bodies [56]. This study did not examine whether TRIM45 could directly act on NLRP3 inflammatory bodies, similar to TRIM50. We did not explore how TRIM45 affects IL-1β and IL-18 mRNA levels. We only revealed the relationship between TRIM45 and NLRP3 in autophagy and mitochondrial dysfunction. We showed that there was an interaction between TRIM45 and Atg5, but we did not explore which domain of TRIM45 could bind to Atg5. There are many core autophagy proteins, and whether there are binding sites between TRIM45 and other proteins remains unclear. Although it has been reported that Atg5 can affect the activation of NLRP3, how Atg5 affects NLRP3 was not examined in this study. Therefore, although this study is helpful for understanding the role of TRIM45 in SAE, the findings need further experimental verification.

## Conclusion

In summary, our results show that knocking down TRIM45 can inhibit microglia-mediated neuroinflammation, protect neurons and slow brain damage caused by SAE. This effect depends on the TRIM45–Atg5 interaction, and so targeting TRIM45 or Atg5 may reverse brain damage and cognitive impairment caused by SAE.

### Supplementary Information

**Additional file 1. Fig. S1.** The expression of TRIM45 in BV2. **Fig. S2.** The expression of TRIM45 in hippocampus. **Fig. S3.** TRIM45 regulates the NLRP3 pathway in an Atg5-dependent manner. **Fig. S4.** The swim velocity and total distance for 4 group in Morris  water maze.

## Data Availability

The data supporting the findings of this study are presented within the manuscript.
