# Supplementary material for: TRIM45 aggravates microglia pyroptosis via Atg5/NLRP3 axis in septic encephalopathy
Source: J Neuroinflammation. 2023 Nov 30;20:284. doi: 10.1186/s12974-023-02959-8 (PMC10688018; doi:10.1186/s12974-023-02959-8)
Supplement: Supplementary file 1 — Additional file 1. Fig. S1. The expression of TRIM45 in BV2. Fig. S2. The expression of TRIM45 in hippocampus. Fig. S3. TRIM45 regulates the NLRP3 pathway in an Atg5-dependent manner. Fig. S4. The swim velocity and total distance for 4 group in Morris water maze. [file 12974_2023_2959_MOESM1_ESM.docx]

**TRIM45 aggravates microglia pyroptosis via Atg5/NLRP3 axis in septic encephalopathy**

**Xuliang Huang^1*^, Changzhou Ye^1*^, Xinyu Zhao^1^, Yao Tong^1^, Wen Lin^1^, Qingqing Huang^1^, Yuhao Zheng^2^, Junlu Wang^1^, Anqi Zhang^1#^, Yunchang Mo^1#^**

*These authors contributed equally: Xuliang Huang and Changzhou Ye.

#Correspondence: zhanganqi0302@163.com and myc1104@wmu.edu.cn

1 Department of Anaesthesia, The First Affiliated Hospital of Wenzhou Medical University, Wenzhou, Zhejiang, China

2 Provincial Key Laboratory of Immune Regulation and Immunotherapy, School of Laboratory Medicine and Biotechnology, Southern Medical University, Guangzhou, Guangdong, China

**Supplementary Materials**

**Supplementary Figures and Legends**

**
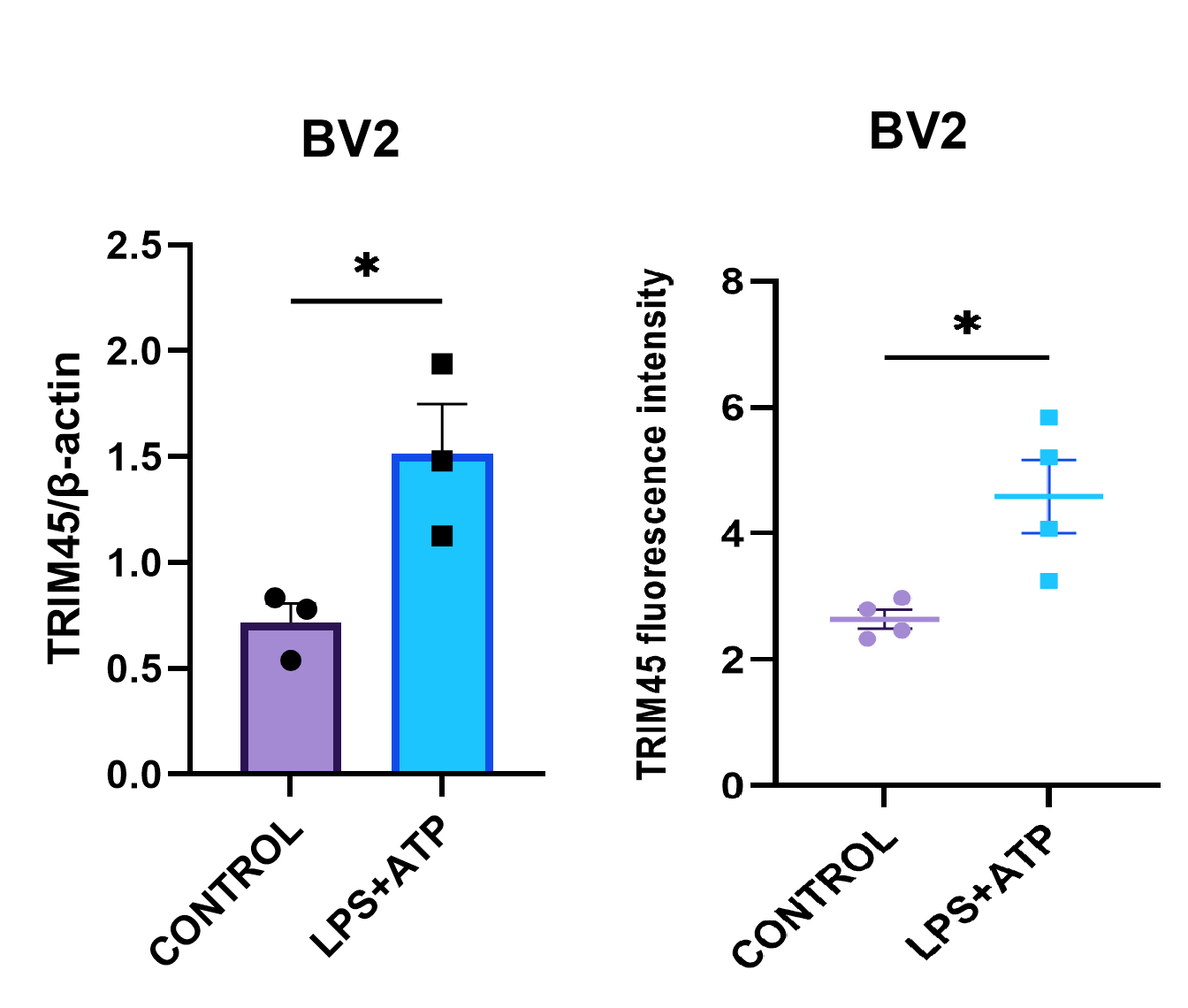
**

**Supplementary Fig. 1** The expression of TRIM45 in BV2. Western blotting and qRT-PCR were conducted to examine the level of TRIM45 in BV2. Data were analysed by unpaired t test. Data are presented as mean ± S.E.M. from at least three independent experiments. *P < 0.05.

**
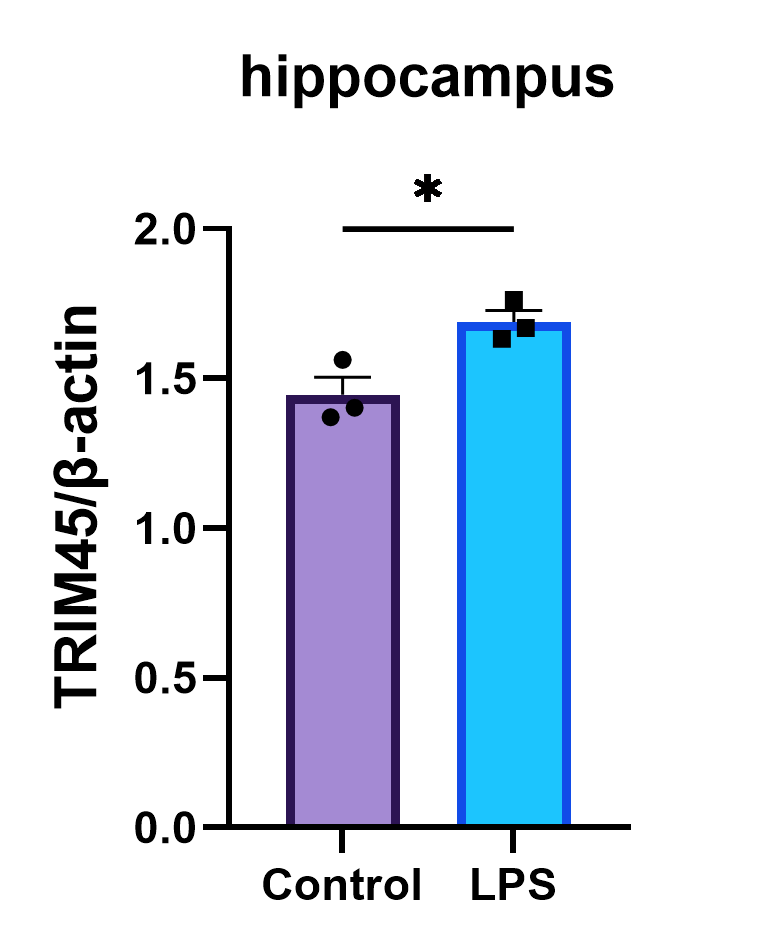
**

**Supplementary Fig. 2** The expression of TRIM45 in hippocampus. Western blotting was conducted to examine the level of TRIM45 in hippocampus. Data were analysed by unpaired t test. Data are presented as mean ± S.E.M. from three independent experiments. *P < 0.05.

**
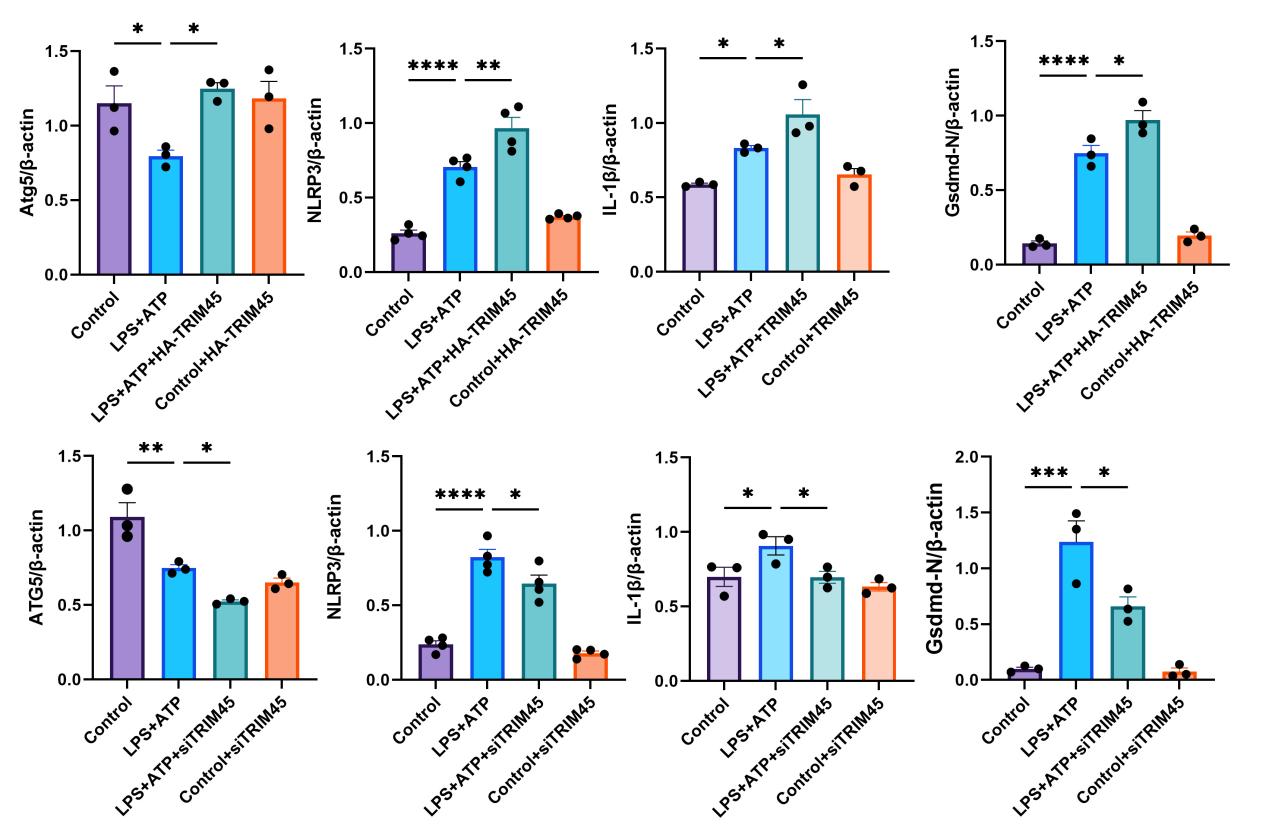
**

**Supplementary Fig. 3** TRIM45 regulates the NLRP3 pathway in an Atg5-dependent manner. We used HA-TRIM45 and siTRIM45 to transfect BV2 cells to analyze the effects of TRIM45 on Atg5 and NLRP3-mediated pyroptosis pathway. Western blotting was conducted to examine the level of Atg5, NLRP3, Gsdmd-N, IL-1β in BV2 cells. Data were analysed by one-way ANOVA followed by Dunnett’s post hoc test. Data are presented as mean ± S.E.M. from at least three independent experiments. *P < 0.05, **P < 0.01, ***P < 0.001 and ****P < 0.0001.


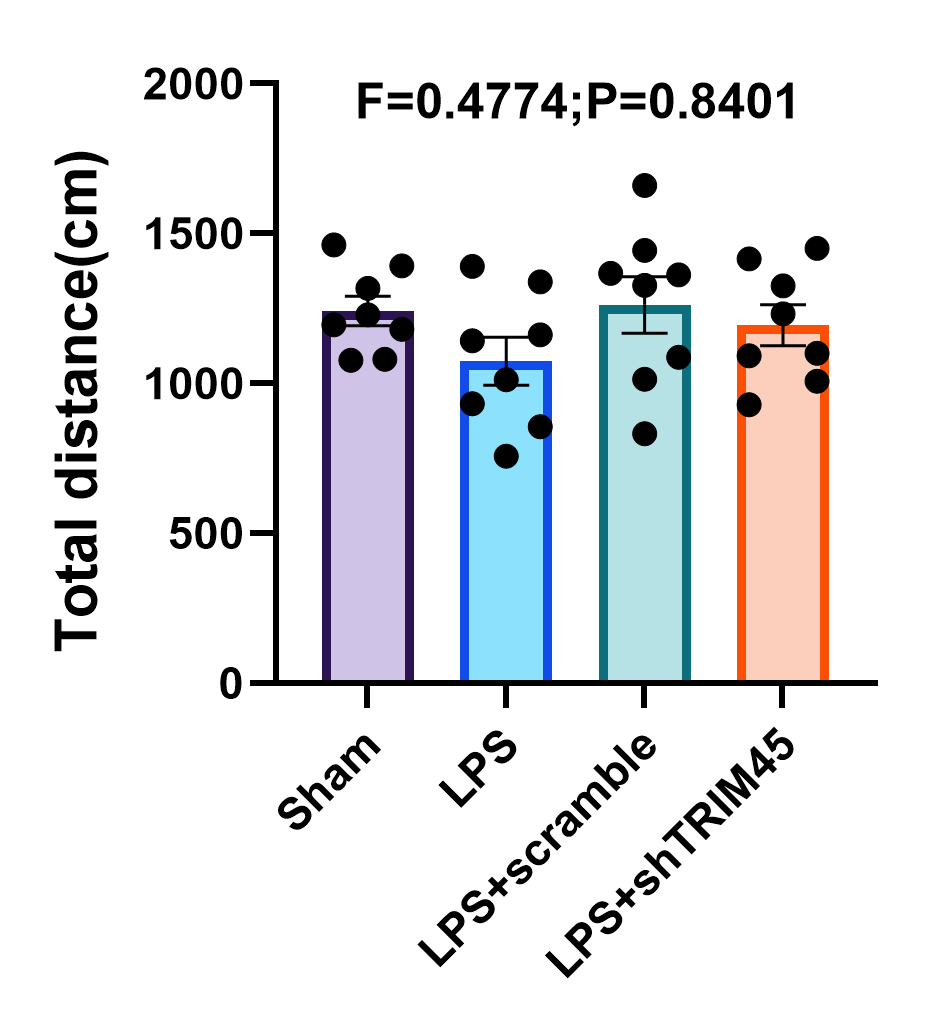

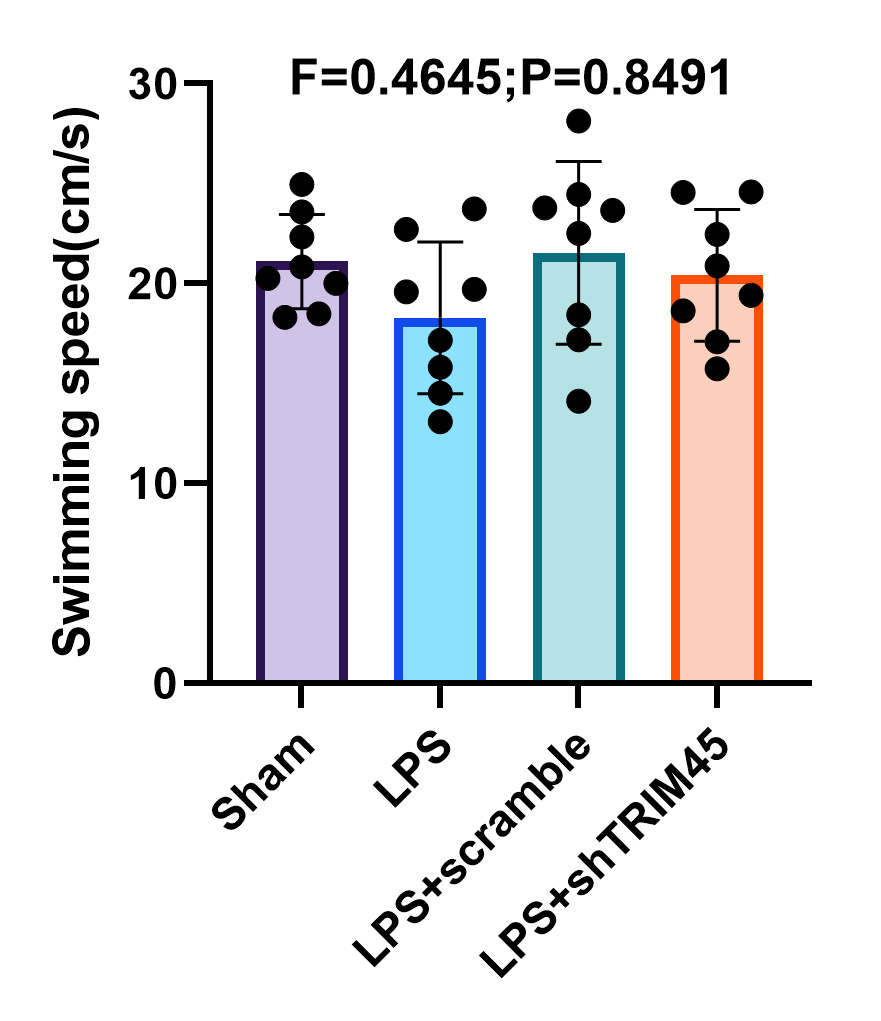


**Supplementary Fig. 4** The swim velocity and total distance for 4 group in Morris water maze. n ≥ 8 per group. Data were analysed by one-way ANOVA followed by Dunnett’s post hoc test. Data are presented as mean ± S.E.M. from at least three independent experiments. P > 0.05.
